# Effectiveness of copper oxychloride coated with iron nanoparticles against earthworms

**DOI:** 10.1038/s41598-024-73794-x

**Published:** 2024-10-05

**Authors:** Tamanna Kumari, Deepak Phogat, Navneet Jakhar, Vineeta Shukla

**Affiliations:** 1 Department of Zoology, Maharshi Dayanand University, Rohtak, Haryana India; 2 Department of Zoology, G.V.M Girls College, Sonepat, Haryana India; 3 Department of Environment Science, Maharshi Dayanand University, Rohtak, Haryana India; 4 Organic Agriculture and Food Systems, University of Hohenheim, Stuttgart, Germany

**Keywords:** Zoology, Environmental sciences

## Abstract

This study examines the potential of iron nanoparticle-coated copper oxychloride in mitigating its toxic effects on earthworms, a key component of sustainable agriculture due to their role in enhancing soil quality and promoting plant growth. While earthworms and their coelomic fluid play a crucial role in enhancing soil health and promoting plant growth. Copper oxychloride, a commonly used fungicide, induces oxidative stress by disrupting antioxidant defense mechanisms in living systems. Through probit analysis, the median lethal concentration (LC50) of copper oxychloride was determined to be 2511.9 mg/kg. Artificial soil was treated with copper oxychloride at 60% and 80% of LC50, but the addition of iron nanoparticle-coated fungicide successfully reduced earthworm mortality to 0%. These findings offer promising insights into protecting non-target organisms from fungicide toxicity while maintaining agricultural productivity. The findings present a potential breakthrough in sustainable agriculture by demonstrating how nanotechnology can mitigate the harmful effects of fungicides on essential soil fauna. The use of iron nanoparticle-coated fungicides not only protects earthworms but also offers a path to maintaining ecological balance and enhancing crop productivity without compromising soil health.

## Introduction

Developed countries produce ~ 80% of the world’s agrochemical formulations. The exaggerated and unchecked practice of these has become a significant bottleneck in the tale of humanity against insect pests, as the resistance spectrum of species is expanding with every passing minute. Extensive usage of these chemicals overshadows their actual motive of practice. Agrochemicals harm the target species and the non-target organisms, which explains secondary pest outbreaks and resurgence. Among all the agrochemicals, fungicide is the second most used in India, with expanded spending by 44.17% from 2014 to 2020^1^. Agrochemicals’ extensive uses (herbicides, pesticides, fungicides, etc.) have drastically affected the soil fauna and biodiversity. These agrochemicals not only affect the soil fauna but also hurt human health. Fungicides (Table 1) are one of the most commonly used agrochemicals in food grains, mainly pulses.

**Table 1:** Change in pesticide consumption, and land use in India^2^.

Element	Value in 2010	Value in 2020	% Change
Pesticide use per area of cropland	0.24 kg/ha	0.37 kg/ha	54% increase
Pesticide use capita	0.03 kg/cap	0.04 kg/cap	33% increase
Agricultural use of fungicide	13055.44 tonnes	20091.58 tonnes	53% increase
Agricultural land area	179,573,000 ha	179,045,100 ha	0.29% decrease
Cropland area	169,234,000 ha	168,669,100 ha	0.33% decrease
Pulse’s area harvested	2,652,980 ha	3,542,159 ha	33.51% increase

Cu-based fungicides like copper oxychloride are being employed to prevent downy mildew in agriculture, specifically in vineyards^3^. Mortality of soil fauna is a close indicator of fungicide toxicity in soil, along with biomass and reproduction. Worms assume a unique role in maintaining soil biophysical conditions like texture, supplement binging, and microorganisms network structure that assists plant growth and development. Even their functions (biological and physiological) depend on the part of the soil they reside in. Depending on soil depth, feeding matter, size, and pigmentation earthworms are divided into different categories that show different body parts modifications. Coelomic fluid released from dorsal pores of earthworms consists of watery fluid, plasma, and many coelomocytes such as amoebocytes, mucocytes, circular cells, and chloragogen cells, which portray a decisive factor in earthworm cellular and humoral immunity for fighting against pathogenic micro-organisms and makes worms a potent therapeutic agent. The coelomic fluid possesses various bioactive compounds, such as proteins, which show a variety of biological functions like antibacterial, anti-cancer, hemolytic anemia, mitogenic, tumorstatic, cytotoxic, and proteolytic activities^4^.

In agronomy, the privileged earthworm’s action aids in checking a few primary affairs in classical farming. In traditional agriculture, aiming for enhanced productivity, the number of nutritional requirements and the risk of nitrate leaching are usually widespread. Indeed, the incorporation of vermicast additives into supplements increases vitamin and mineral utilisation efficacy while decreasing nitrate leaching fates. Additionally, achieving huge crop profits suggests a great degree of resistance to disease and pests^5^.

Along with enhancing awareness against chemical pollutants, diverse research is also going on divergent techniques for their remediation. Amid all of them, nanotechnology aced them in the last century. Using less-managed, perennial, sustainable, easily accessible, widely distributed, eco-friendly plant parts extract can reduce the cost of isolating microorganisms, the cost of culture media, and the danger of contamination by half. Since the dawn of civilization, plants have fulfilled a variety of functions, the most recent of which is their superior cost-effectiveness in producing nanoparticles as opposed to microbes. *Azadirachta indica* for green synthesis, plant materials such as seeds, leaves, bark, etc. are used. Terpenoids, alkaloids, flavonoids, and other phytomolecules play a key role in the creation of plant-mediated nanoparticles (NPs). Oddly, the majority of phytochemicals are water-soluble, which has led to the development of inexpensive, non-hazardous solvents^6^. In addition to preventing site contamination, these phytochemicals’ biodegradability as capping agents has no impact on the adsorption of NPs on active site surfaces^7^. A quick breakdown of active substances reduces residue on food, improves safety for users (humans) and non-target organisms, and prevents pest resistance and comeback. Due to their nutraceutical benefits in preventing numerous lifestyle-related disorders like diabetes, hyperlipidemia, digestive issues, cephalgia, and others, plants are preferred over synthetic antioxidants in the food sector^8,9^.

## Materials and methods

### Chemical for testing

Copper oxychloride, commercially available in the market, was procured after seeking proper permission. Label name-Blutoxx (Copper oxychloride 50% WP), active agent: Shivalik Crop Science (P) Ltd, an ISO 9001 and 14,001 certified company (Table 2).

**Table 2:** Physiochemical properties of copper oxychloride (PubChem, NCBI; PPDB).

Parameters	Copper oxychloride
Solubility	1.19 mg/l in water and 11 mg/l in toluene at 20 °C
Vapour pressure	0.00001 mPa at 20 °C
Structural formula	Cl_2_Cu_4_H_6_O_6_
Purity of test chemical	50%
Molecular weight	427.14
Melting point	decompose before melting
Degradation temperature	240 °C
Colour	Green
Odour	Odourless
Biodegradability	Not biodegradable
WHO classification	II (moderately hazardous)

### Iron nanoparticle synthesis

Fresh and healthy leaves from the medicinal plant were taken from the University’s herbal garden in Rohtak, Haryana, India. The samples were carefully washed multiple times with running water, then and dried in air. With the use of an electric grinder, the air-dried leaves were crushed to a fine powder. 50 g of dried leaf powder was boiled in 1000 ml of distilled water for one hour at 80 °C. Using Whatman’s no. 1 filter paper, filtrate was obtained. The filtrate was then stored at 4 ℃ for further experimentation. Ferric precursor and plant extract were combined in a 1:1 ratio with regular stirring at 50–60 °C^10,11^, and the mixture was then centrifuged at 10,000 rpm for 40 min. For 24 h, prepared NPs were dried in the oven.

### Coating of fungicide with nanoparticles

An 8:2 v/v water: ethanol solution was prepared with an LC50 concentration of fungicide to reach specific molarity^12^. Suitable amount of particles were dispersed in an 8:2 v/v water: ethanol solution to obtain the same molarity. Nanoparticles were sonicated in a water bath with a sonication. A NaOH solution was used to bring the reaction’s pH to 7–8. After being combined, both solutions were kept in reflux for a few hours. Finally, the ethanol evaporated, and the coated nanoparticles were dried in an oven at 65 °C for 24 h.

### Analytical characterization of NPs and coated NPs

Various analytical techniques were employed to characterize NPs for morphology, size, purity, elemental composition, and charge. Instantaneous conformity of bio-reduction of Fe^3+^ to Fe^0^ was read by UV-Visible spectroscopy and change in colour and pH, XRD, EDX, and FESEM analysis of the NPs were characterized at IISER, Pune. Photoluminescence spectra, FT-IR studies, and zeta analysis was done at ACIL, Maharshi Dayanand University, Rohtak, Haryana, India.

### Test organisms

In laboratory conditions, it was simple to culture or handle *Eisenia fetida* and showed results in a short period. The life cycle was short (7–8 weeks), with a high reproduction rate and rapid growth. The worm is known for its easy cultivation of organic waste in a short time. Because they assist in mineral recycling, they are being employed as bioindicators of soil toxicity. These earthworms have been commonly used in the past 50 years^13,14^. The European Union and the OECD have approved it for toxicity tests.

### Procurement of earthworm

The study was conducted on vermicomposting worm, *Eisenia fetida*. The worms have high reproductive rates and are easy to culture on various media in the laboratory. *Eisenia fetida* was procured from Bhoojevan organics Pvt. Ltd. Location-Najafgarh, Delhi. Worms were reared by OECD Guidelines no. 207 (1984), ISO (1993,1998)^15–17^, and tropical artificial soil was used as a medium.

### Calculation of LC50 value of fungicide to earthworm

The culture was maintained according to OECD guidelines 207, 1984. *Eisenia fetida* thrived in artificial soil amended with cow manure. Ecotoxicological assays were carried out in Tropical Artificial Soil, as India (28.8768° N, 76.6211° E; 597 mm annual rainfall) falls in the tropical range, an adaptation of Garcia (2004) of artificial OECD (1984)^18^. After the TAS was mixed and homogenized, its acidity was corrected where necessary with CaCO_3_.

### Estimation of rate of application of fungicide and fungicide-coated NPs

Once LC50 was evaluated, artificial soil was prepared, and the fungicide and fungicide-coated NPs were added at different doses. The artificial soil was prepared per OECD guidelines 207 (1984), and the tests were conducted to evaluate the effect of different doses of fungicide on various parameters of *Eisenia fetida.* Again, the artificial soil test was conducted to estimate the application rate of fungicide-coated NPs to nullify the LC50. The different treatments of fungicide-coated NPs were prepared where the concentration of LC50 of fungicide and the NPs spike the artificial soil. Each treatment had three replicas, with ten healthy, well-clitellated worms added for each experiment setup.

#### Experimental design

Post-acclimatization animals were randomly selected into 5 Groups, each containing 10 animals (Table 3).

**Table 3:** Experimental design for the rest of the objectives.

S. No.	Treatment	Effects studied after	Group name	Replicates
1	Control	Week 1, week 2, week 3, week 4	Group A	3
2	60% LC50	Week 1, week 2, week 3, week 4	Group B	3
3	80% LC50	Week 1, week 2, week 3, week 4	Group C	3
4	1: 0.75 LC50 and NPs	Week 1, week 2, week 3, week 4	Group D	3
5	0.85: 1 LC50 and NPs	Week 1, week 2, week 3, week 4	Group E	3

### Comparative metal analysis in exposed earthworm

Firstly, the worms were sacrificed by extrusion of their body fluids into 50% ethanol. After drying at 70 °C overnight, samples were fragmented (< 0.2 mm). Samples were heated for two hours at 120 ℃ after being digested with 15 ml HNO_3_ and 2 ml H_2_O_2_^19^. Before measurement, the volume of the solution was made up to 25 ml. AAS measured copper concentrations in samples.

### Assay to check oxidative stress

#### Enzyme extraction

Agar gel was prepared for worm gut clearance. 1.5% agar gel was prepared with distilled water. After refrigerating the agar in the jars, the gel was cut into small pieces. The homogenate was centrifuged separately at 10,000 rpm at 4 °C for 10 min after gut-cleaned earthworms were pestled in ice-cold mortar with 50 mM potassium phosphate buffer (1:8 w/v; pH 7). The supernatant was used for enzyme activity and protein determination^20,21^.

#### Protein determination

The supernatants were subjected to total protein estimation with Bradford’s assay and BSA as a standard. The absorbance of the dilution was recorded at 595 nm within minutes of mixing^22^. The protein content of each homogenate sample was then calculated from the standard curve of BSA.

#### ROS content analysis

Earthworms were homogenized at 4 °C using ice-cold phosphate buffer (100 mM, pH 7.4). Centrifuge at 3000 rpm at 4 °C for 5 min. The supernatant was centrifuged at 20,000 rpm at 4 °C for 20 min. DCFH-DA and mitochondria suspension were mixed with a certain amount of PBS buffer and mixed to make the final reaction liquid and heated in the water bath (37 ℃). The fluorescence intensity was measured after terminating the reaction by mixing 200 µl HCl. The excitation wavelength was fixed at 490 nm and emission at 520 nm.

#### POD (guaiacol peroxidase EC 1.11.1.7)

Peroxidase (POD) activity was measured by adopting the guaiacol mode^23^. A reaction mixture of 3 ml was prepared by mixing 100 mM potassium phosphate buffer (pH 6), 30% H_2_O_2_, and guaiacol in concentrations of 50 ml, 0.038 ml, and 0.056 ml, respectively. After the addition of enzyme extract and absorbance reading was taken at 470 nm.

### Comparative total phenolic content of exposed earthworm

Earthworms were placed in a Petri dish containing extrusion medium (5% ethanol, 95% saline, 2.5 mg/ml EDTA, 10 mg/ml guaiacol glycerol ether; pH 7.3) to stimulate the spontaneous release of coelomic fluid. After removing worm bodies and centrifuge extrusion medium at 8000 rpm at 4 °C^24,25^; the samples were exposed to the Folin-Ciocâlteu reagent procedure^26^. 50 µl of the sample was blended with 3 ml of deionized water, followed by 250 µl of FC reagent. A few moments later, 20% sodium carbonate was added to the mixture. The samples were incubated in the dark. Absorbency was read at a wavelength of 765 nm. The results obtained were expressed as Gallic acid equivalents (GAE) using Gallic acid as standard.

### Histological analysis of worms

#### Hematoxylin and eosin staining

For H and E staining the section mounted over the lysine-coated slides was air-dried for 1 h, followed by dehydration in 95% ethanol for 2 min. The slides were then immediately dipped for about 10–15 min in 4% hematoxylin solution. The slides were water-washed for 30 s to wash away any excess hematoxylin stain. Water washing was followed by dipping in 1% acid alcohol solution and again water was washed under a running tap for minutes. The slides were then subjected to 2% eosin stain for 5–10 min, followed by water wash for removing the excessive stain. The stained sections were subjected to gradual alcoholic dehydration in 70% and 100% ethanol, respectively, for 1 min each. The stained and dehydrated slides were xylene-cleared and air-dried followed by mounting with DPX and coverslips. The sections were observed under the microscope for morphological alterations^27^.

#### Statistical analysis

All data collected of the mean value of 3 replicates of control, and all the treatments groups exposed worms were subjected as mean ± SD for analysis by “One-way and Two-Way ANOVA followed by Tukey’s multiple comparisons test were performed using GraphPad Prism^28^. The mean and standard deviation were also calculated using MS Excel 365. The significance of utilizing ANOVA single factor as the statistical tool for analysis of the results was to compare the mean value of more than two groups, control, and two different amendments. Two-way ANOVA Tukey’s HSD test for multiple comparisons was performed for multiple comparisons. All the data in this study are presented as mean ± SD. Two-way ANOVA with correction was invoked to evaluate the relation among various treatment groups keeping a significance level equivalent to p < 0.05. Data are presented as-ns(p > 0.05), *(p < 0.05), **(p < 0.01), ***(p < 0.001) and **** (p < 0.0001).

A graphical presentation of the difference in various content of organisms between control (Group A) and treated groups (60% LC50 (Group B), 80% LC50 (Group C), 1: 0.75 LC50 and NPs (Group D), and 0.85: 1 LC50 and NPs (Group E) over a 28-days exposure. Alphabet represented significant differences between the groups; *p < 0.05; a presented a significant difference from Group C to Group D and E exposed within a week; b presented a significant difference from Group A to all the rest exposed within a week; c denotes the significant difference of a group week-wise (moreover the doses showing all the three statistically differences are represented by d i.e., a, b, c = d) via two-way ANOVA.

## Results and discussion

### Characterization of NPs and coated NPs

#### Analytical characterization

##### pH

The instantaneous transformation of plant extract solutions from different shades of yellow to black instantly by blending iron precursor confirmed the development of Fe-NPs. Analysis of freshly prepared solution of FeCl_3_ was 1.46. The plant extract had a pH of 5.30, which changed to 1.64 after mixing with the ferric chloride solution. 1.39 was the pH of nanoparticles after 20 h.

#### Optical behaviour

##### UV-visible analysis

**Fig. 1:**
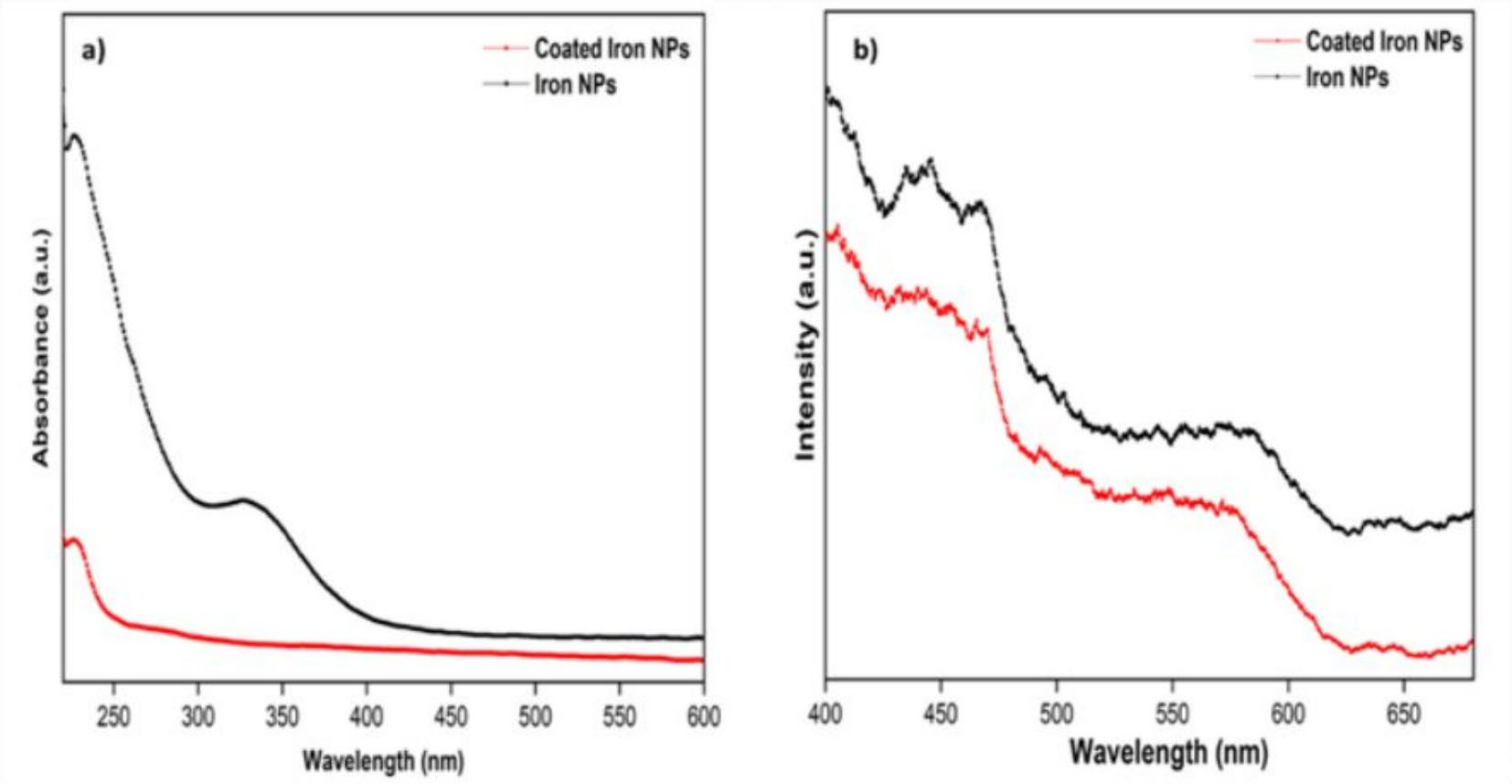
(**a**) UV-Visible analysis of NPs and coated NPs against different wavelengths (**b**) Photoluminescence spectra of NPs and coated NPs.

UV-Visible analysis, used as a confirmatory tool by resonating with metal photons, showed a strong plasma resonance in UV-Visible portion of the electromagnetic spectrum. 0.3 ml Fe-NPs were diluted with water to make the total volume of 3 ml. Figure 1a showed an extensive and size-dependent peak in the UV-Visible range of 216–265 nm, confirming Fe-NP formation. The results obtained were in good agreement with Pattanayak and Nayak (2013).

##### Photoluminescence analysis

Recent discoveries confirmed photoluminescence as a defining characteristic of nanoparticles that mainly depended on the surface states’ shape, size, synthesis conditions, and energetic positions. It was a light-emitting phenomenon from visible to near-infrared region. Excitation mechanisms used in quantum dots ranged from bound excitons to surface plasmons in metal nanoparticles. The solid-state fluorescent property of d6 metal complexes was studied considering their excellent luminescent properties. When excited at 350 nm at room temperature, the PL spectra of Fe-NPs synthesized are depicted in Fig. 1b. The NPs showed a strong luminescence band located in the blue region at 460–470 nm^29^. Luminescence originated because of the emission existence of defects and vacancies of multiple atoms present. At 350 nm, an electron excited from the valence band to the conduction band leaving behind a hole, resulting in a sharp peak. The small intensity and band gap difference between green synthesized and chemically synthesized NPs may be because of plant extract biomolecules on the Fe-NPs surface.

##### XRD analysis

**Fig. 2:**
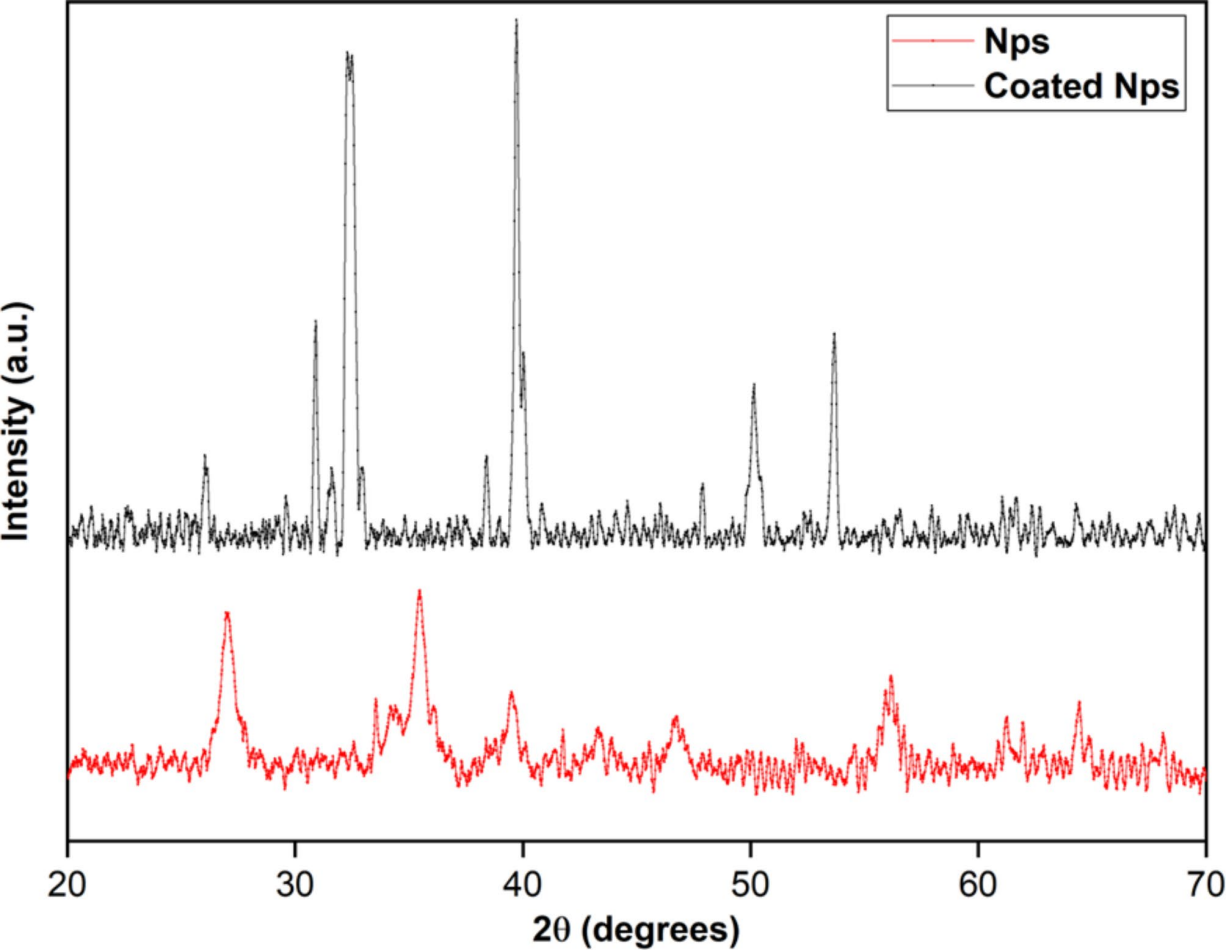
XRD spectra for powdered material. The scanning range used for the sample analysis was 20°≤ 2θ ≥ 70°. The broadening of the peaks indicated that the particles were of nanometer scale. The characteristic peak shows the formation of a crystalline phase.

The XRD analysis of samples was done in a range of 20° to 70° of 2θ using an anode of Cu-K_α_ with a scanning rate of 2°/minute explained in Fig. 2. Utilizing the peak broadening profile size of iron NPs was calculated using Scherer’s formula,

$$D = \frac{{0.94\uplambda }}{{\upbeta \text{Cos} \uptheta }}$$

Where λ = 1.5418 Å K = 0.94, a dimensionless shape factor, θ diffraction angle, and β is the line broadening at half of the maximum intensity in radians of respective peaks. The strain and dislocation density were calculated by

$$\upvarepsilon = \frac{{\upbeta \text{Cos} \uptheta }}{4}$$

$$\updelta = \frac{1}{{{\text{D}}^{2} }}$$

The findings were consistent with those of Ebrahiminezhad et al. 2018; Das and Dhar; 2022^30,31^. More crystallinity of NPs was proportionate to more intensity of XRD peaks. Distinct peaks in the XRD pattern confirmed the existence of secondary metabolites that governed the purity and stability of NPs. NPs prepared were of crystallite character; measurable dimensions, requiring fewer plant extracts and stirring time. XRD data of nanoparticles showed the presence of iron and copper through the peak at 2θ at 43.35 and 32.22. The size of NPs calculated from Scherrer’s equation ranged from 52 to 64 nm, averaging 63.63 nm. other parameters calculated from XRD data of NPs are 0.24 nm d spacing, -0.03 strain, and 2.46*10^14^ lines/m^2^ dislocation density.

##### Morphological and structural analysis of NPs.

**Fig. 3:**
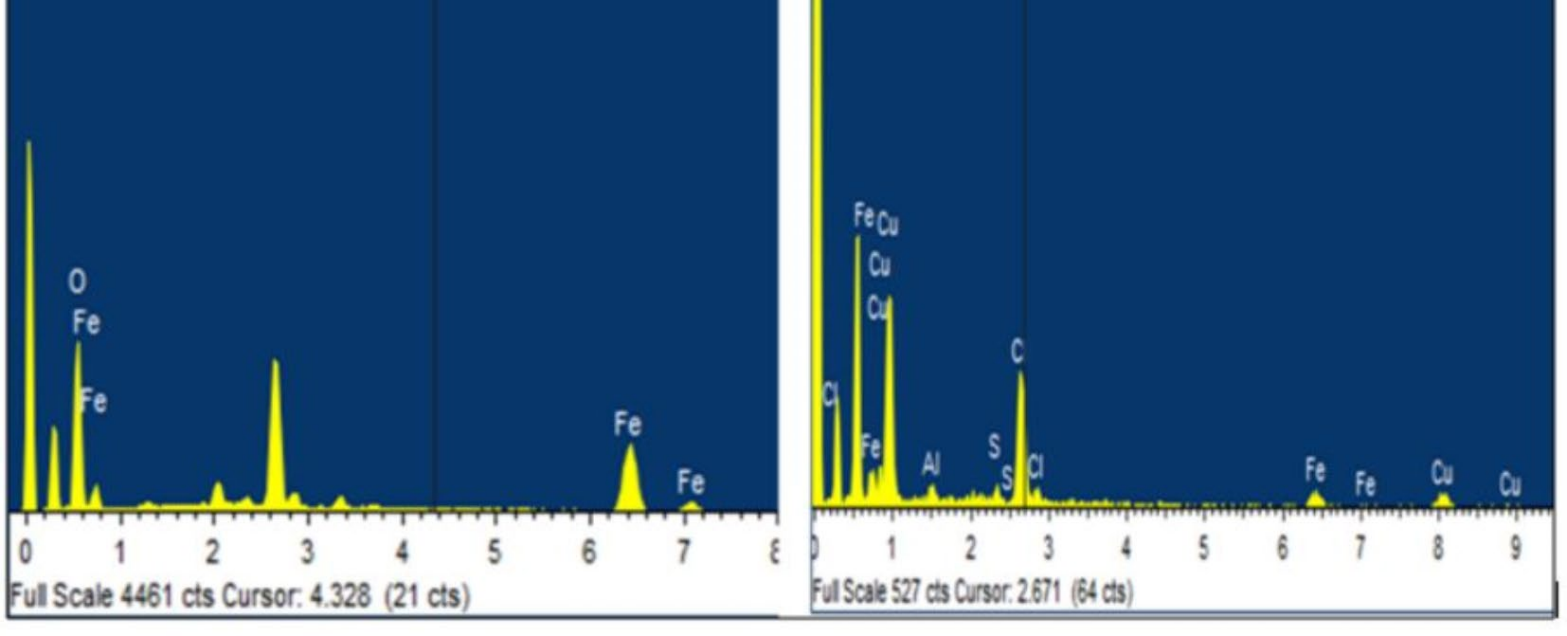
EDX analysis of Fe-NPs and coated NPs.

FESEM-EDX showed that piercing X-rays energize electrons resulting in voltage signals (Fig. 3). EDX analysis gave a clear-cut idea about the iron % in the sample and its oxide formed in the synthesis of NPs.

**Fig. 4:**
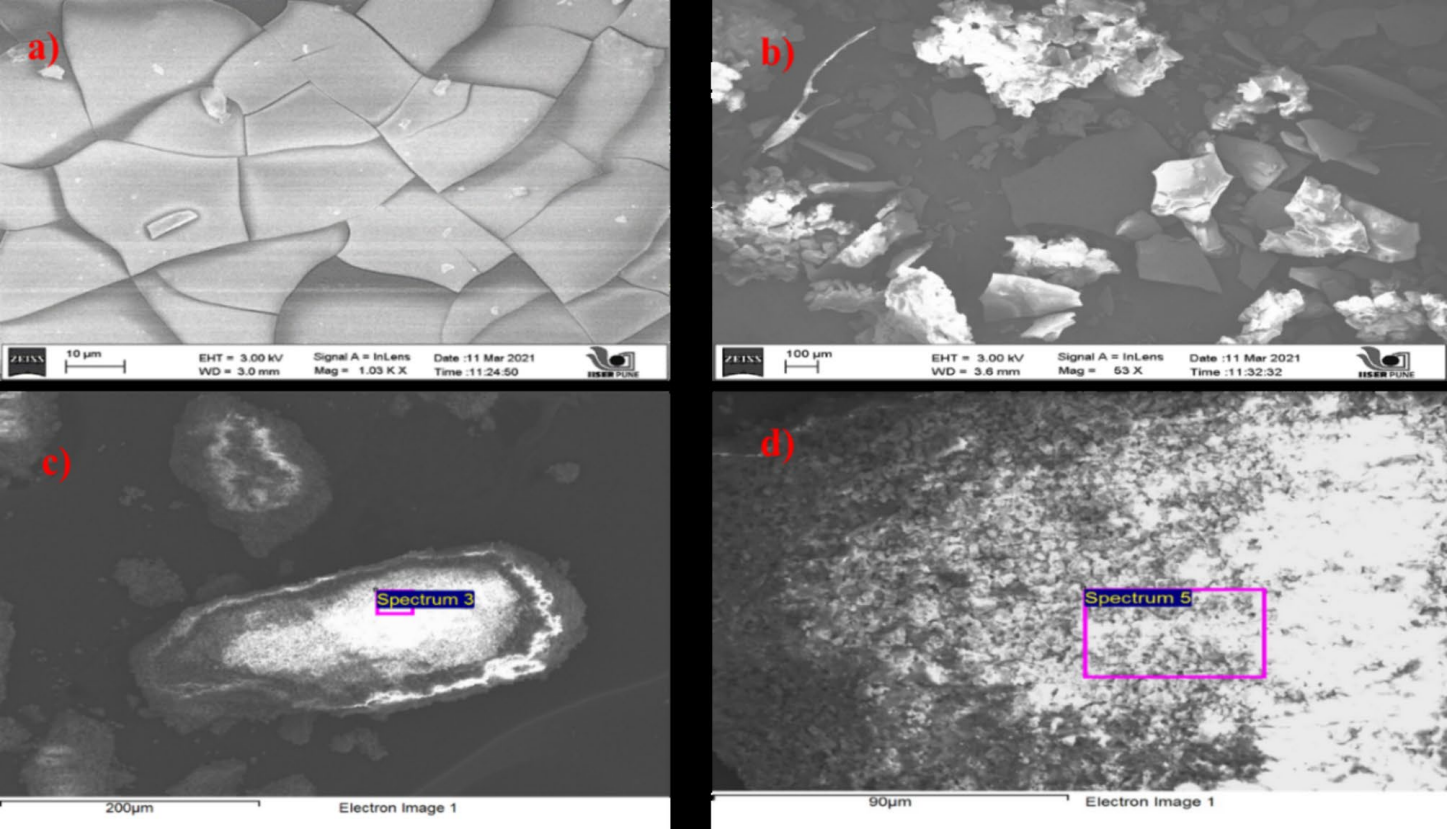
FESEM image of (**a**) Fe-NPs at 10 μm magnification (**a**) Fe-NPs at 100 μm magnification (**c**) coated NPs at 200 μm magnification (**d**) coated NPs at 90 μm magnification.

Reflected X-rays fitted iron having peaked at 0.6, 0.8, 6.4 and 7.0 keV from elements’ surface. Strong copper signals were recorded in coated NPs at 0.9, 8 and 8.9 keV^32^. Because of non-uniform particle size and voids, results demonstrated irregularities. The EDX analysis showed the purity of NPs (Table 4). The results of FESEM were given at two different magnifications, as shown in Fig. 4. Some peaks of impurities were also observed in coated NP’s EDX spectra, which were present in the substrate.

**Table 4:** EDX analysis of NPs and coated NPs.

Element	Iron NPs (weight%)	Coated Iron NPs (weight%)
Al	-	4.97
S	-	4.39
Cl	-	46.24
Fe	51.65	15.10
Cu	-	29.30
O	48.35	-
Total	100	100

##### Fourier transform infrared analysis

Both the synthesized compound underwent FT-IR analysis for functional group pinpointing. The study focused on a 4000 –400 cm^− 1^ mid-infrared region. FT-IR shorten the data acquisition time and output was improvised for signal-to-noise ratio.

**Fig. 5:**
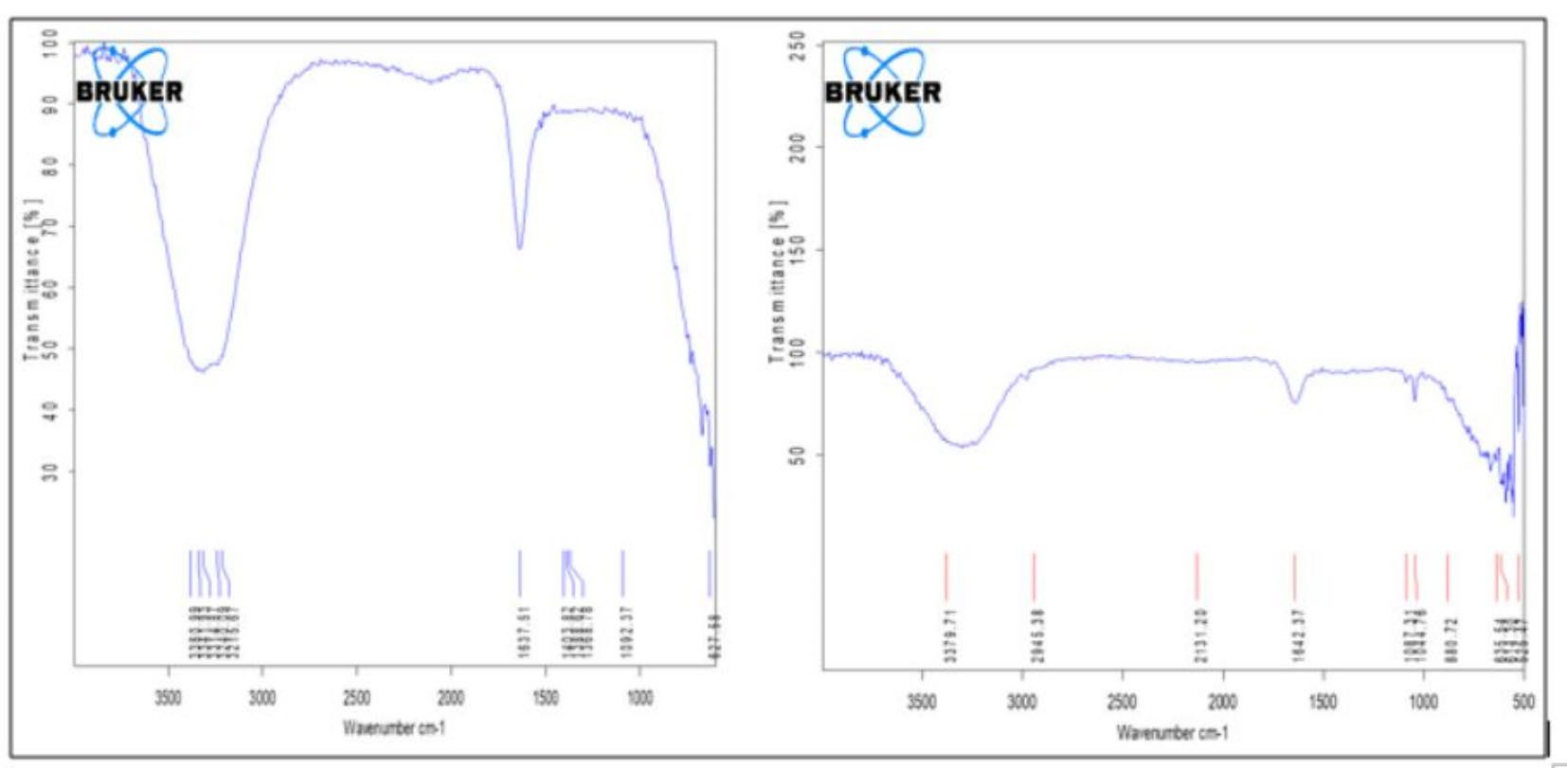
FT-IR analysis of Fe-NPs and coated nanoparticles.

Absorption bands of FTIR were utilized to spot the compound functional group employed in reducing the precursor, as absorption occurs only if the infrared light frequency matched the bond’s vibrational frequency. All the other absorption bands other than Fe-O showed that the polyphenols acted as a capping and reducing agent along with causing steric or electrostatic hindrance that brought in enhanced stability (Fig. 5; Table 5).

**Table 5:** Absorption band obtained through FT-IR spectrum.

Sample	Wavelength (cm^− 1^)	Functional group
Iron NPs	3381	O-H group
	1637	C=C aromatic ring stretching
	1388	C-N stretching vibration of aromatic amines
	1092	C-O stretching vibrations
	627	Fe-O
Coated Iron NPs	3380	O-H stretching
	2945	C-H stretching
	2131	Cu-H stretching
	1087	C-O stretching
	1044	C-O stretching
	880	Cu-O-H stretching
	635	Fe-O stretching
	525	Cu-O stretching

##### Zeta sizer analysis

**Fig. 6:**
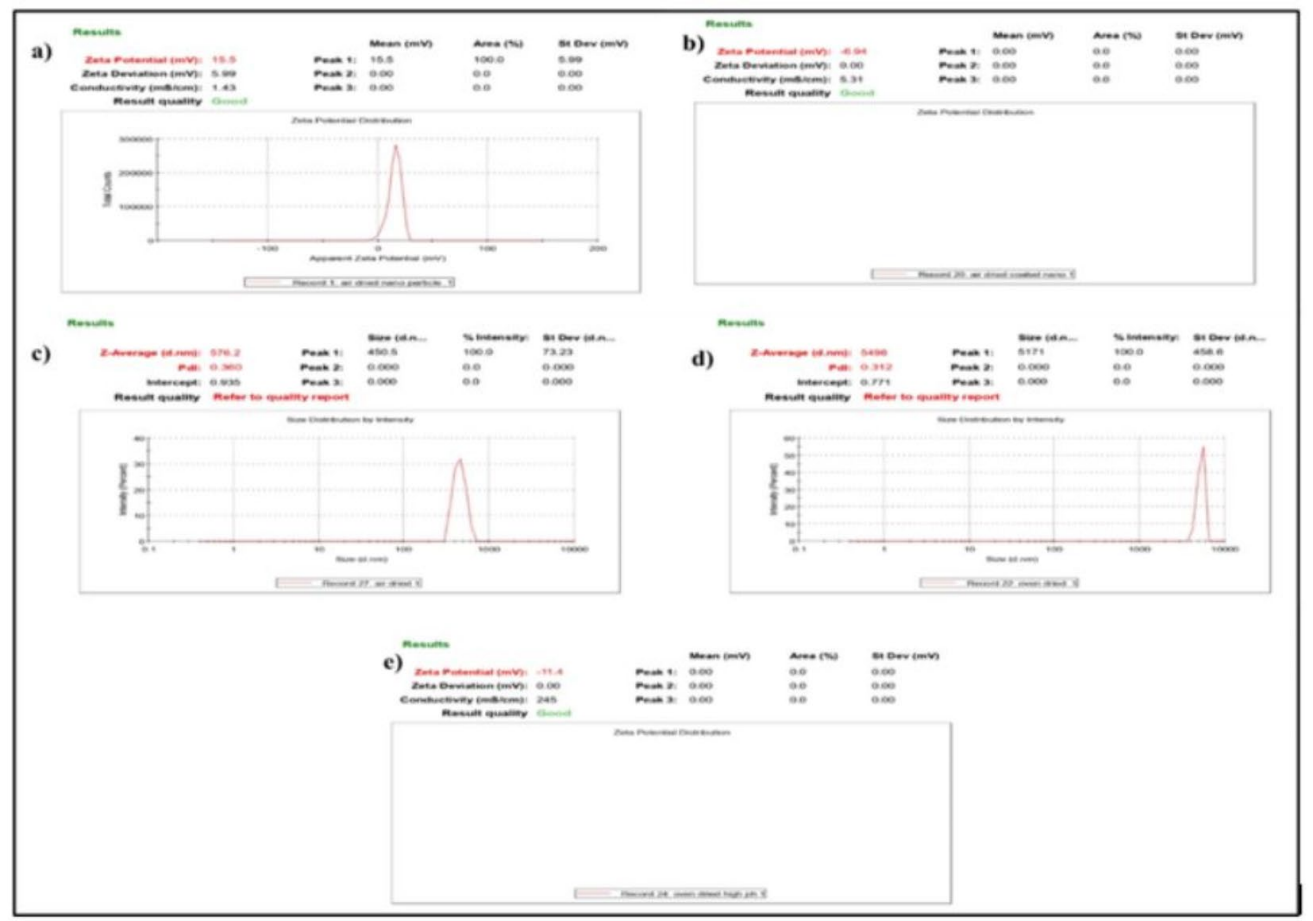
Zeta sizer analysis (**a**) Zeta potential of Fe-NPs in acidic pH; (**b**) Zeta potential of coated NPs; (**c**) Zeta size analysis of Fe-NPs; (**d**) Zeta size analysis of coated NPs; (**e**) Zeta potential of Fe-NPs in basic pH.

Dynamic Light Scattering did Zeta sizer analysis at an incident angle of 90°. pH was a characterizing circumstance for zeta analysis in aqueous sap. As the pH value increased, negative values of zeta potential changed to cations at lower pH due to the protonation of functional groups like amide, carboxyl, etc. The zeta potential value of ˃60 mV reproduced excellent stability, followed by the reasonable value of 40–60 mV. In contrast, a value of ˂30 mV illustrated agglomeration by Van der Waal’s force of attraction. Zeta analysis of freshly prepared NPs showed a positive value of potential (Fig. 6), which shifted to negative upon increasing pH and varied dimensions due to agglomeration. A polydispersity index that varied between 0 and 1 is a crucial criterion for reading the size distribution width. For colloidal particles, the dispersity index shifts towards 0, indicating monodisperse particles. In our case, it ranged more toward 1, which meant more agglomeration.

Through the reducing ability of secondary metabolites, the active principle reduced precursor FeCl_3_ to Fe^0^. The identified secondary metabolites acted as insect attractants for pollinators, provide pigmentation, antioxidants protecting against ROS, etc. Phenolic molecules protected the cell or organisms which they inhabited against ROS, DNA damage, carcinogens, cell ageing factors, etc.

###  Characterization of earthworms

**Fig. 7:**
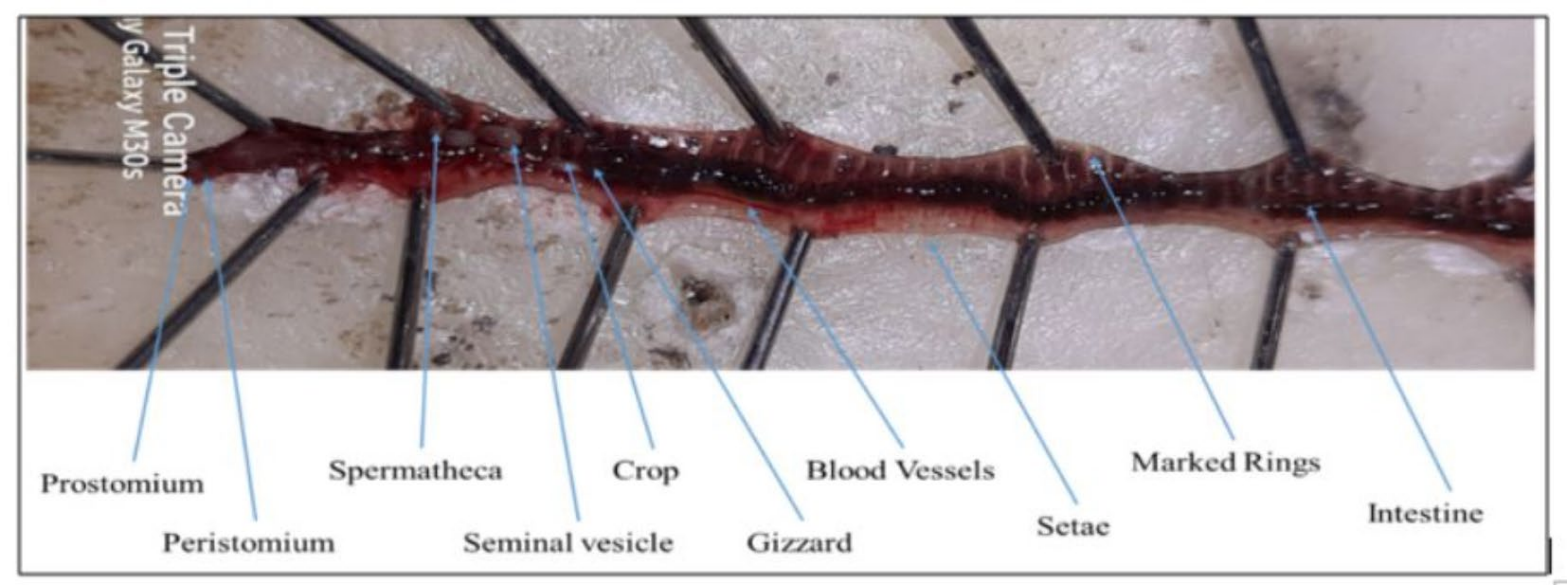
Anatomy of *Eisenia fetida*.

They are small, around 3–4 inches long, with reddish-purple body colour. The “rings” around worms are known as segments and closely paired setal arrangements. To constrain open slits in soil, they pose a projection on the anterior surface to cover the mouth, called prostomium. *Eisenia fetida* is hermaphroditic in nature, with the position of clitellum in reproductively active worms from 26 to 32 segments. Reproductively active worms mate and exchange sperms, which are stored in the spermatheca (Fig. 7). Later, the clitellum hardens, and the development of the cocoon starts, which is laid in moist soil.

The incubation period ranged from 10 to 44 days and the number of juvenile emerging from the cocoon range from 1 to 9^33^. The cocoons are lemon-shaped and are light yellow from the start, getting progressively brownish as the worms inside become adults. These cocoons are noticeable to the naked eye. Worms have developed nervous, circulatory, digestive, excretory, muscular, and reproductive systems. Lubricating mucous emitted by skin glands encourages worms to travel through the soil and balances out tunnels and castings.

### Estimation of LC50 of copper oxychloride for *Eisenia fetida*

For estimating the LC50, six concentrations in a geometric series were used. The concentration selected was 100 mg, 200 mg, 400 mg, 800 mg, 1600 mg, and 3200 mg/kg soil. The mortality was noted at each concentration on the 14-day interval, and the test temperature was 22°± 2 °C. Testing was done in continuous light. The LC50 of copper oxychloride on *Eisenia fetida* was calculated using the log dose/probit regression line method^34^. This represented the dose-mortality relationship for earthworms. A median lethal concentration (LC50) of copper oxychloride was computed using probit analysis and was found to be 2511.9 mg/kg.

y = mx + c (y is 50% mortality, m is the slope of log10 concentration- probit curve, c is the intercept and x is the log concentration).

LC50 = 2511.9 mg/kg.

### Estimation of rate of application of fungicide and fungicide-coated NPs

After calculating the LC50 of copper oxychloride, it was evident that the application rate as an amendment also had to be estimated to nullify the mortality at the LC50 from 50% to below or 0%. For this, the fungicide-coated nanoparticles were added in different doses in the artificial soil (Tables 6 and 7).

**Table 6:** Mortality of Eisenia fetida for fungicide versus the rate of application of fungicide-coated nanoparticles (expressed as mean).

S. No.	Treatment	Mass of the treatment (mg)	% of NPs w.*r*.t copper oxychloride	Alive animal	Whether Selected/ not selected
1.	Control			10	
2.	LC50	2511.9	0	5	
3.	1: 1 LC50 and NPs	2511.9 + 939	37.38	8	
4.	1: 0.75 LC50 and NPs	2511.9 + 704.25	28.03	10	Selected
5.	1: 0.85 LC50 and NPs	2511.9 + 798.15	31.77	10	
6.	0.75: 1 LC50 and NPs	1883.91 + 939	49.84	10	
7.	0.85: 1 LC50 and NPs	2135.09 + 939	43.97	10	Selected

**Table 7:** Correlation analysis of mortality of Eisenia fetida for fungicide versus the rate of application of fungicide-coated nanoparticles.

Parameter	% NPs and dead animal
R Square	0.68
Standard Error	1.27
P-value	0.04
F	8.80
Statical significance	Yes

The treatment’s selection criterion was the minimum concentration of the catalyst that could show the maximum response and the other one showing the maximum catalyst load of nullifying the LC50.

The mechanisms between nanoparticles and fungicides were the electrostatic effect, hydrogen bonding, van der Waals interaction, hydrophobic interaction, ligand exchange, and cation bridging. The structural and surface heterogeneity of nanoparticles made these interactions quite sophisticated. The photoluminescence spectra of bare and coated NPs, clearly showed a spectrum with a peak at 530 nm. Absorption bands of coated NPs confirmed the presence of iron and copper as their functional groups. In addition to iron strong copper signals were recorded in coated NPs at 0.9, 8 and 8.9 keV. Along with this, the coating was better demonstrated in FESEM data, which clearly shows the deposition on nanoparticles. The bare nanoparticles clearly showed a crystalline nature, which turned out to be globular and amorphous when coated.

After this, the zeta analysis was in which potential shift by 22.44 mV and size by 4921.8 nm. The dispersity index shifted towards 0 (coated nanoparticles-0.312) for colloidal particles, indicating monodisperse particles. In the case of the bare nanoparticles, it ranged more towards 1 (0.360), which meant more agglomeration.

### Comparative metal analysis in both exposed earthworm

After the calculation of LC50, organisms were exposed to different sublethal doses as for various experiments. The method utilized for assessing metal content was the tissue digestion method. Both one-way and two-way ANOVA were performed to compare the effects of treatments on the copper content of earthworms. One-way ANOVA (Tukey’s HSD test for multiple comparisons) revealed that there was a statistical difference in the copper content of groups to control with F (4, 15) = 10.26 with a significant p-value of 0.0003.

When all the treatment mean values of 4 weeks were compared, control with 60% LC50, control with 80% LC50, 80% LC50 with 1: 0.75 LC50 and NPs, and 80% LC50 with 0.85: 1 LC50 and NPs showed a statistically significant difference in worms with a p-value of 0.0382, 0.0009, 0.0023, 0.0017.

**Fig. 8:**
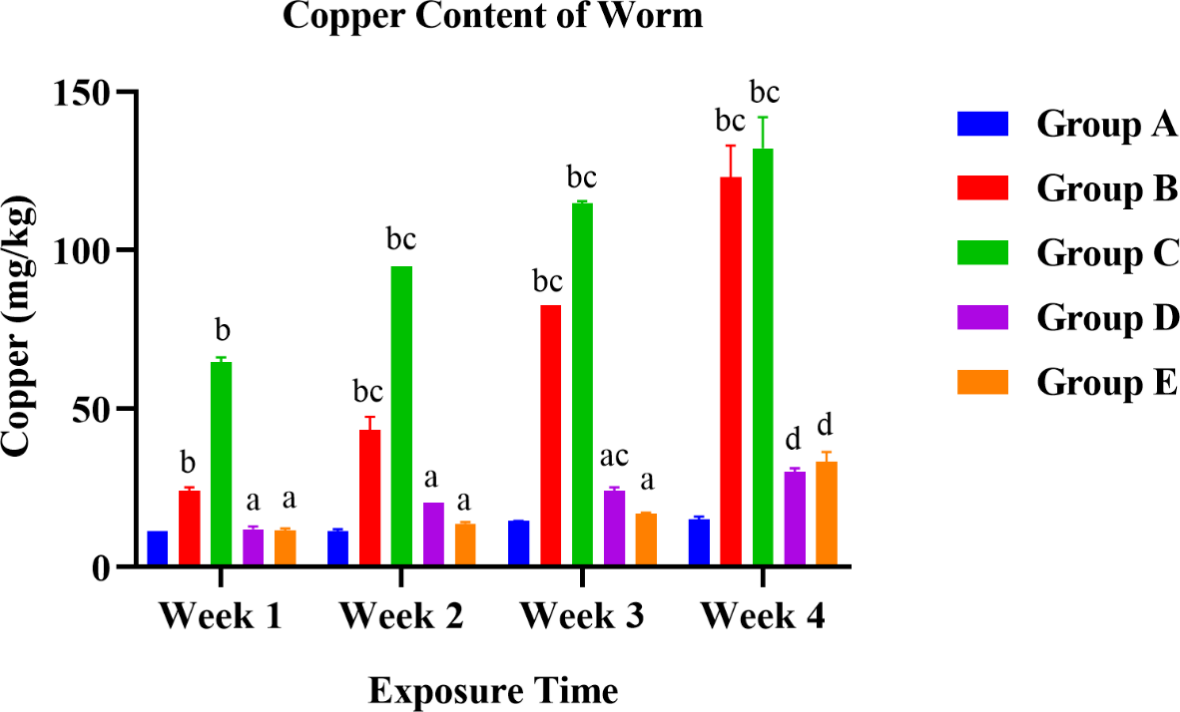
A graphical presentation of the difference in copper content of worms.

The bioaccumulation of the harmful chemicals in the earthworm body has been discussed as a mitigating factor for entering these excess chemicals into the food chain via plants. They are an integral part of the complex food web. The biomagnification is increasing day after day. Figure 8 summarizes the mean copper content of the control and treated groups. The copper content of worms between treatment and control groups showed a significant difference except for the week 1 mean value of 1: 0.75 LC50 and NPs (Group D) and 0.85: 1 LC50 and NPs (Group E). Presumably, the pollutants mainly bioaccumulate in earthworms via epidermal absorption and feeding^35,36^. Earlier investigations have demonstrated that Cu toxicity and accumulation in earthworms’ bodies included the calciferous glands through chloragogenous tissue^37,38^. As exposure time had increased in fungicide-treated groups, the body’s copper content was also increasing linearly with outer copper external exposed concentrations. While copper content increased in 1: 0.75 LC50 and NPs (Group D) and 0.85: 1 LC50 and NPs (Group E) treated groups, it did not increase as much as in the fungicide-treated groups. Increased size of pollutants for epidermal absorption, reduced solubility in soil pore water, reduced mobility of ions, and high soil organic matter content reduce the bioavailability of pollutants to the worm. However, there was a sharp increase in copper content as the exposure time increased from weeks 3 to 4, mainly by 0.85: 1 LC50 and NPs (Group E) and 60% LC50 (Group B) with 51.5% and 33.3%.

### Assess oxidative stress in exposed earthworm

#### ROS content analysis

Both one-way and two-way ANOVA were performed to compare the effects of treatments on the ROS content of earthworms. One-way ANOVA (Tukey’s HSD test for multiple comparisons) revealed that there was a statistical difference in the ROS content of groups to control with F (4, 15) = 18.04 with a significant p-value of < 0.0001. When all the treatments mean values of 4 weeks were compared, control with 60% LC50, control with 80% LC50, control with 1: 0.75 LC50 and NPs, 60% LC50 with 0.85: 1 LC50 and NPs, and 80% LC50 with 0.85: 1 LC50 and NPs showed a statistically significant difference in worms with p-value < 0.0001, 0.0002, 0.0039, 0.0099, 0.0006.

**Fig. 9:**
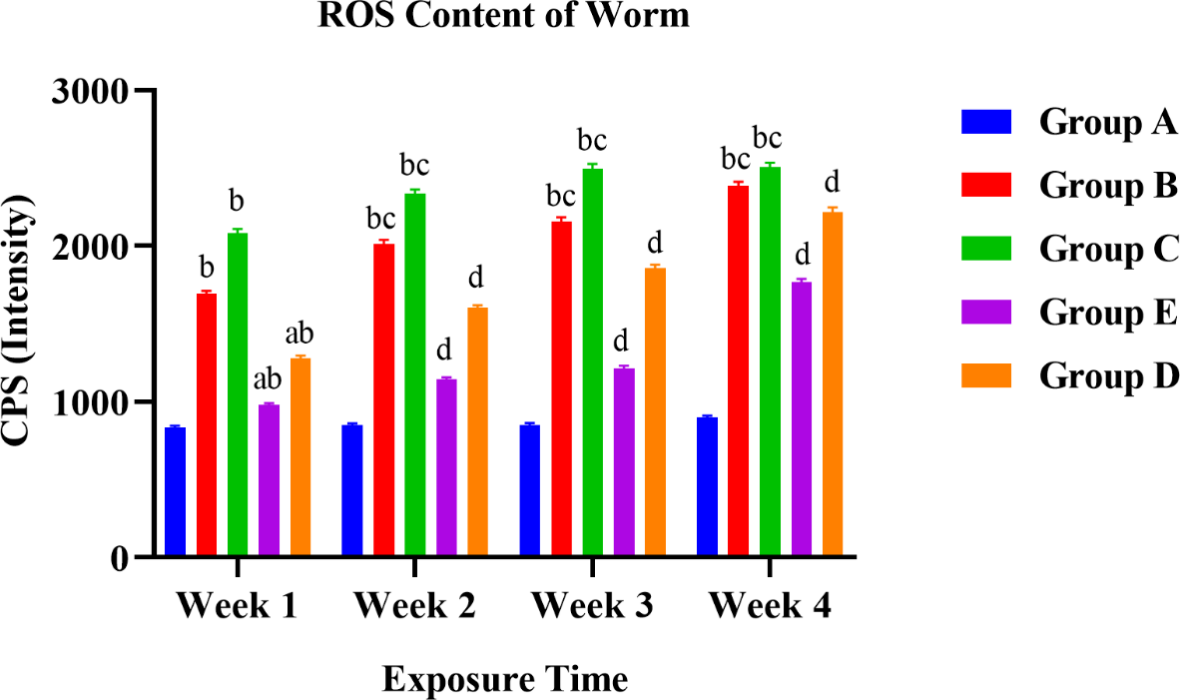
Graphical representation of ROS content in worms.

#### POD enzyme activity

Both one-way and two-way ANOVA were performed to compare the effects of treatments on the POD levels of earthworms. One-way ANOVA (Tukey’s HSD test for multiple comparisons) revealed that there was a statistical difference in the POD level of groups to control with F (4, 15) = 38.45 with a significant p-value of < 0.0001. When all the treatment mean values of 4 weeks were compared, the control with 60% LC50, control with 80% LC50, control with 1: 0.75 LC50 and NPs, and control with 0.85: 1 LC50 and NPs groups showed a statistically significant difference in worms with p-value < 0.0001, < 0.0001, < 0.0001, 0.0002.

**Fig. 10:**
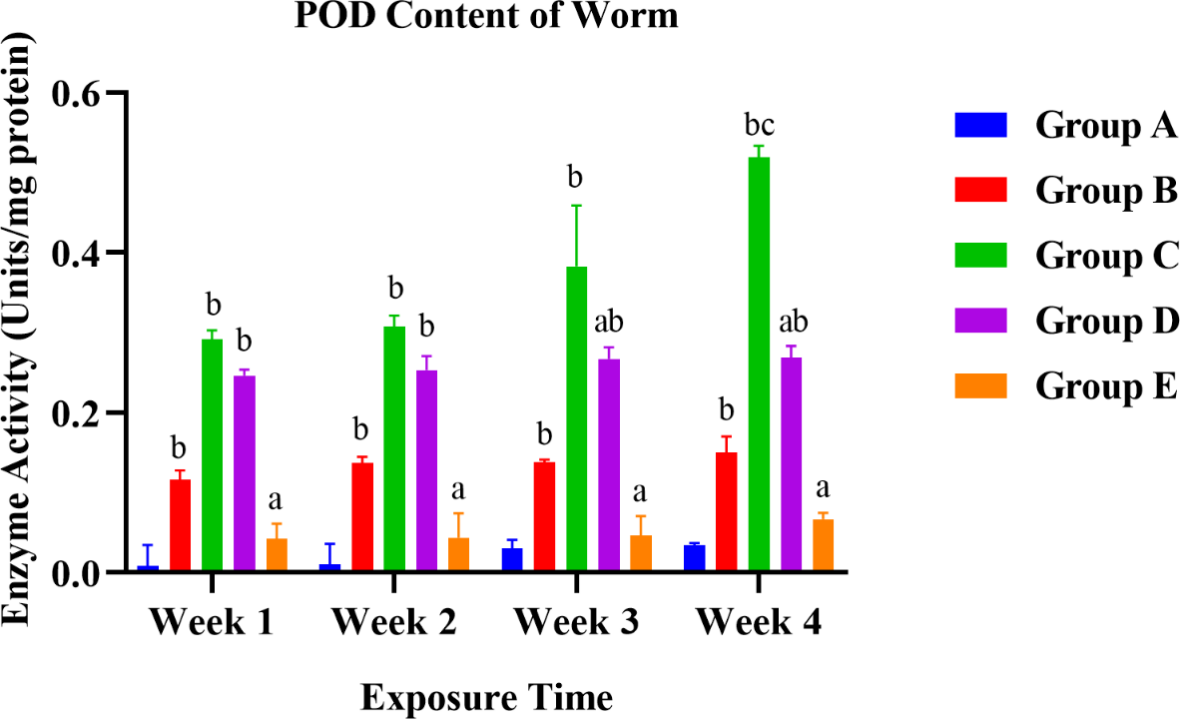
Graphical presentation of POD enzyme activity in worms.

The levels of worms between treatment and control groups showed a significant difference except for POD, which decreased the mean value of the control (Group A) to 0.85: 1 LC50 and NPs (Group E) from weeks 1 to 4. Figures 9 and 10 showed an upregulated ROS and POD content in worms with a dose-response relationship. The most significant rise in ROS content was seen in worms at 80% LC50 (Group C) and 0.85: 1 LC50 and NPs (Group E). A similar trend in ROS level was observed for worms when exposed to PCB77 and nZVI; pentachloronitrobenzene (PCNB), and ZnO nanoparticles^39,40^. ROS and POD expressions are overexpressed when organisms are exposed to stressful conditions. Earlier reported copper content results showed the stressful conditions in the culture medium. They were responsible for surpassing their average expression levels to protect vital biomolecules against oxidative stress and afford antioxidant defence mechanisms^39^.

### Comparative total phenolic content of both exposed earthworm

Both one-way and two-way ANOVA were performed to compare the effects of treatments on the total phenolic content of earthworms. One-way ANOVA (Tukey’s HSD test for multiple comparisons) revealed that there was a statistical difference in the total phenolic content of groups to control with F (4, 15) = 30.04 with a significant p-value of < 0.0001. When all the treatment’s mean values of 4 weeks were compared, control with 60% LC50, control with 80% LC50, control with 1: 0.75 LC50 and NPs, 60% LC50 with 1: 0.75 LC50 and NPs, 60% LC50 with 0.85: 1 LC50 and NPs, 80% LC50 with 1: 0.75 LC50 and NPs, 80% LC50 with 0.85: 1 LC50 and NPs groups showed a statistically significant difference in worms with p-value < 0.0001, < 0.0001, 0.0330, 0.0232, 0.0004, 0.0002 < 0.0001.

**Fig. 11:**
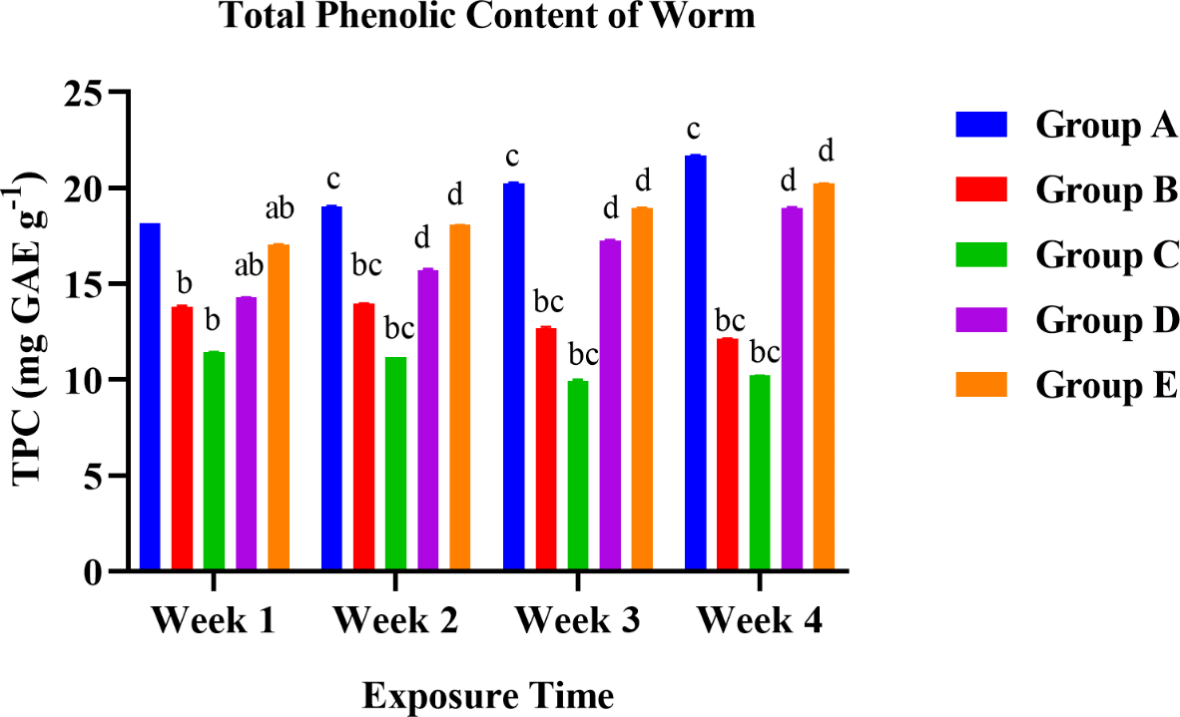
Graphical presentation of total phenolic content in worms.

As shown in Fig. 11, the total phenolic content was reported as mg/g of the test samples in gallic acid equivalent (GAE). Results demonstrated the significant difference in total phenolic content of worms between control (Group A) and fungicide-treated groups and 1: 0.75 LC50 and NPs (Group D). The higher propensity of phenolic compounds to chelate heavy metals can be partly attributed to their biological and antioxidant actions.

### Histological analysis of worms

#### Effect on the body wall of *Eisenia fetida*

**Fig. 12:**
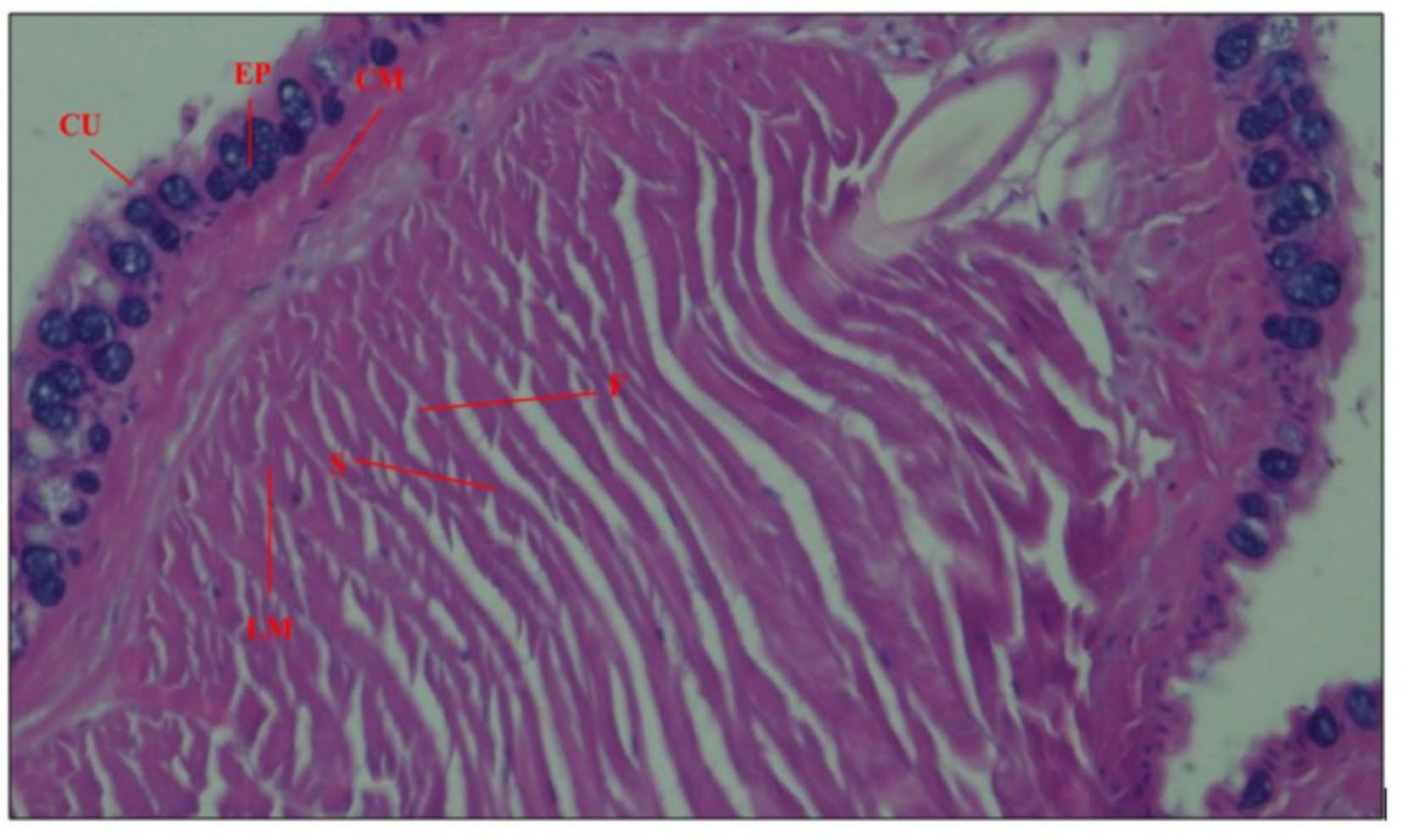
The diagrammatic representation shows the structure of the body wall [Outer Cuticle (CU), Epidermis (EP), outer circular muscle layer (CM), inner longitudinal muscle layer (LM), with arrangement of fibrils (F) around radial connective tissue septum (S)].

The control worms showed normal morphology. The epidermis and cuticle were intact. There was a normal distribution of different types of cells in the epidermis. There was a clear compartmentalization of the epidermis. Some mucous cells contained mucous, while others were empty. There was a normal arrangement of the muscle fibres, both outer circular and inner longitudinal muscle layers were intact and showed a normal arrangement of fibres and fibrils (Fig. 12). The coelomic peritoneum was also intact in the control worms.

Upon fungicide treatment, ulcerous erosion of the external body wall was noticed. A proliferation of the mucocytes was noticed, and in addition disintegration of transverse muscles was also noticed. In addition, fibrosis in the circular muscle fibres was also noticed.

**Fig. 13:**
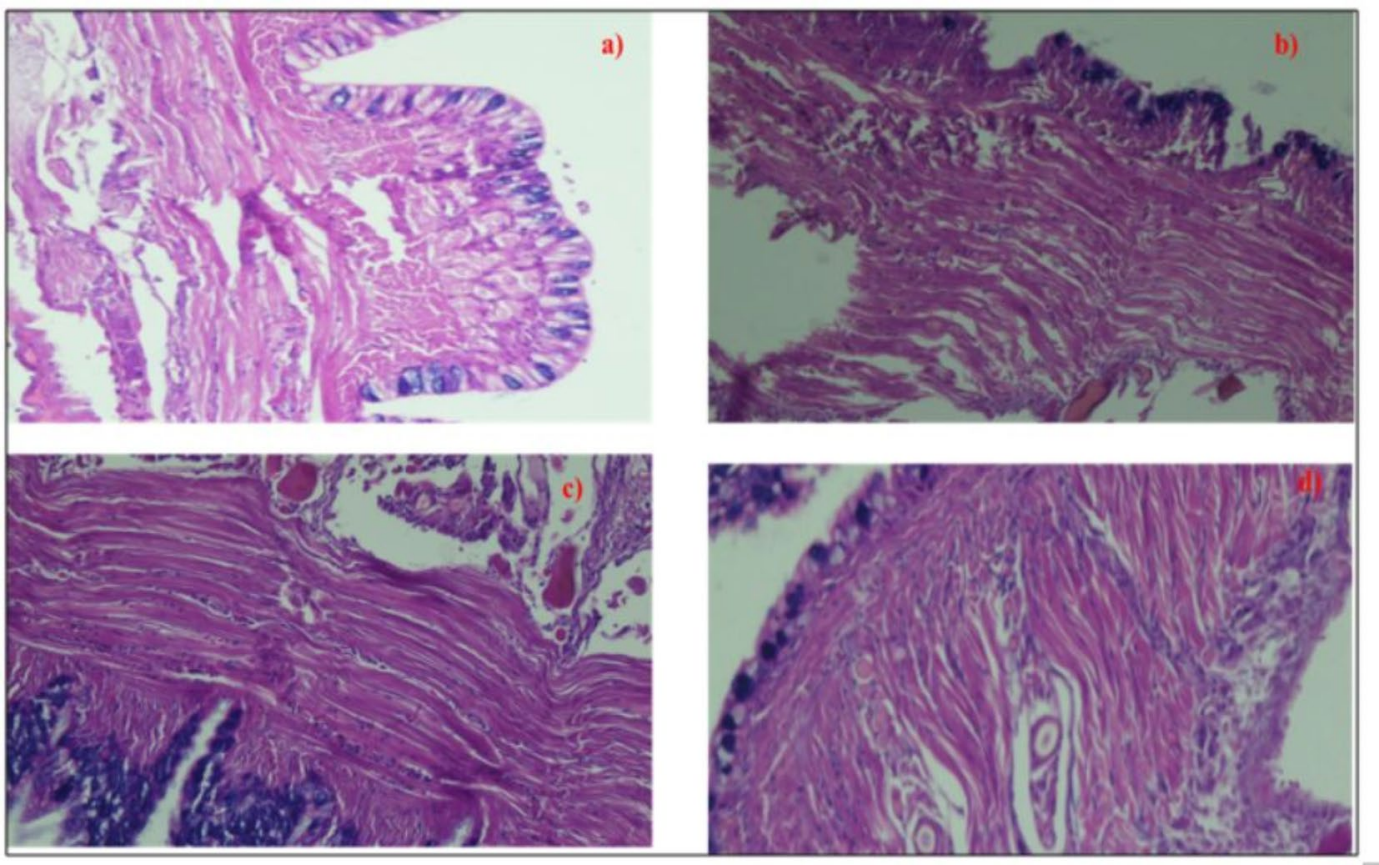
Diagrammatic presentation of the body wall of worm (**a**) 60% LC50 (**b**) 80% LC50 (**c**) 1: 0.75 LC50 and NPs (**d**) 0.85: 1 LC50 and NPs after an exposure period of 28 days.

Hypertrophy of the apical part of the epidermis and slight loss of structural integrity of the epidermis were seen. Besides the epidermis, the circular and transverse muscles were also disintegrating at places, and separation of the muscle layers was also noticed. The structural integrity of the epidermis was lost, and muscle fibres separation increased significantly. There was a complete loss of normal arrangement of fibres and fibrils. Under exposure of worms, total disintegration of the muscle fibres was observed, and the radial connective tissue septum and fibrils arranged around it could not be differentiated. The muscle layers seemed to have lost their normal condensed state (Fig. 13).

The epidermis was also not smooth and appeared ridged. At the time of exposure to coated NPs, thickness in the epidermis was observed, with most of the mucous cells appearing to have been emptied. Thickening of the epidermis was a common change observed in the worms exposed to different sub-lethal doses, though thickening did not necessarily correlate with increased mucous glands.

#### Effect on the gut of *Eisenia fetida*

**Fig. 14:**
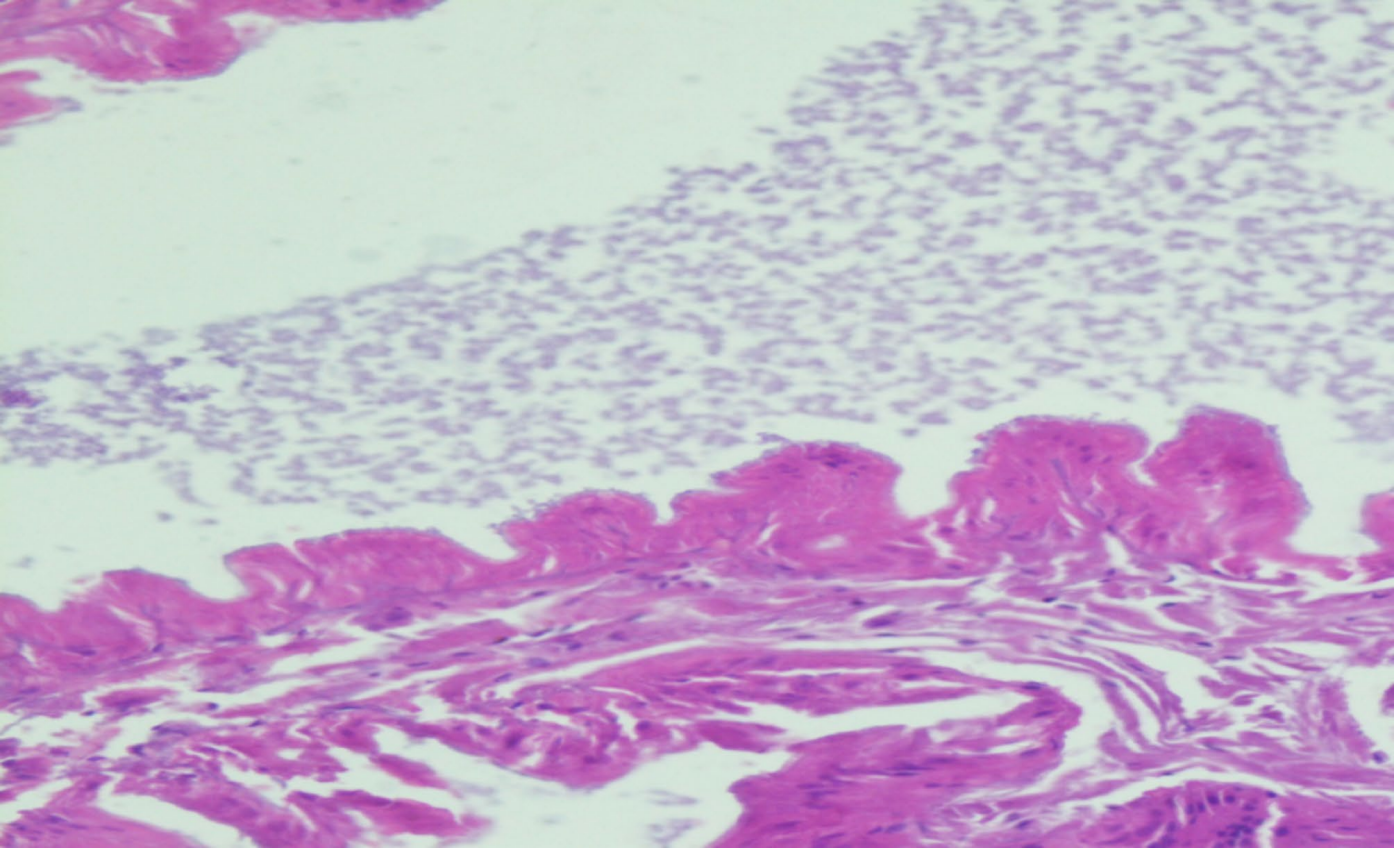
Histological analysis of intact villi of earthworm.

**Fig. 15:**
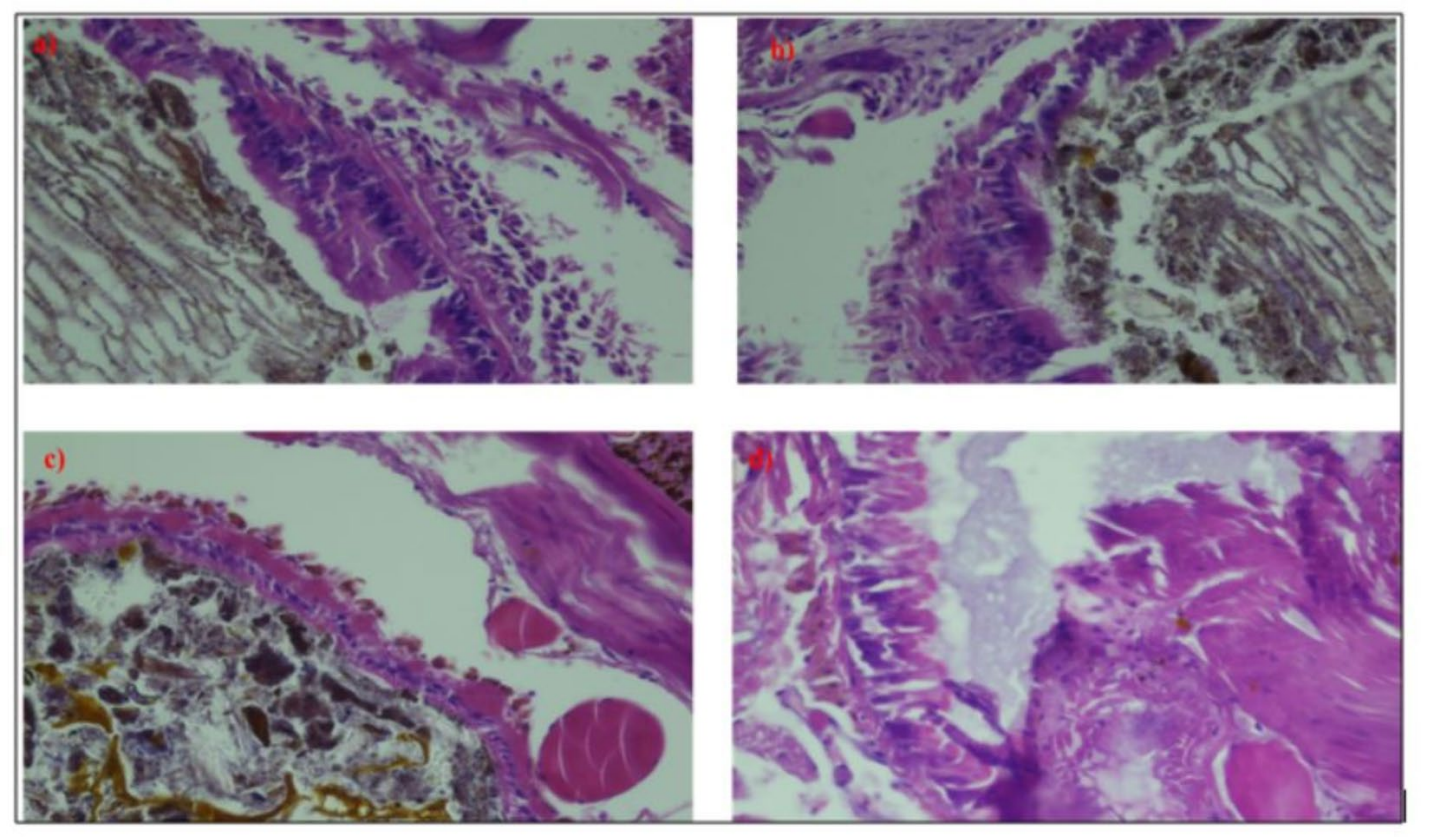
Diagrammatic presentation of the gut of worm (**a**) 60% LC50 (**b**) 80% LC50 (**c**) 1: 0.75 LC50 and NPs (**d**) 0.85: 1 LC50 and NPs after an exposure period of 28 days.

The guts of the control epithelium were densely packed, however upon treatment, the density of the gut epithelium was found to be decreased in the post-clitellum region and the loosening was more visible. The loosening was also seen in the post-clitellum region, and the epithelium was nearly disintegrating at several points. Treatment did not only compromise the integrity of post-clitellum regions but the effects were also observed in the pre-clitellum and the clitellum region. The loosening of the gut epithelium in the clitellum region was mild in the post-clitellum region, the loosening effect was moderate. There was significant erosion to the intestinal villi as well. Under exposure, there was significant vacuolization observed in the tissue. The appearance of vacuolization pointed towards the degradation of the tissue layer. They appeared as pyknotic nuclei in the tissue when exposed. It was due to the condensation of the chromatin in the cell which pointed towards cell death or apoptosis being initiated in the cell (Figs. 14 and 15).

The treatments were found to have a lot of effect on the physiology and biochemistry of the *Eisenia fetida* earthworms. Do these changes manifest in the anatomy of the earthworm? The microtomy of the earthworm was performed to register these changes. The anatomical changes were very important to estimate the physical manifestation of the ill effects of treatments. Anatomical changes in various animals were reflective of tissue damage. The histopathological changes can be used as a marker for exposure to toxic materials. Moreover, histopathological changes can be used to predict the toxic effect of a toxicant^41^.

Earthworms dwell in the soil and the cuticle of the worms remains in contact with the soil. The effects of the toxic chemicals in the soil would directly interfere with the physical integrity of the earthworm’s cuticle and body wall. The microtomy slides of the control earthworms showed normal histology of the body wall of *Eisenia fetida*. At 60% LC50 (Group B) of fungicide pyknotic nuclei, fused villi, cellular debris, and a reduced lumen were found. Whereas, in the amendment, no intervillous space was found. There were fused villi, a slightly reduced lumen, and a few pyknotic nuclei. On the other hand, earthworms exposed to treatments 0.85:1 LC50 and NPs (Group E) with nanoparticles showed a better organization of villi. The villi were structurally maintained without pyknotic nuclei. The proper intervillous space and a normal lumen were found. At high sublethal doses, the villi were disintegrated and fused, with no intervillous space, and many pyknotic nuclei were seen. Due to necrotic activity in worms exposed to high doses, there was pigmentation with cellular debris.

The outer gut wall of earthworms is surrounded by chloragogen cells, which are derived from the inner coelomic epithelium. The chloragogen cell performs various physiological functions including excretion metabolism, synthesis, and storage of fat and glycogen. These cells work like the liver of earthworms, hence playing a significant role in metabolism. In the control group, these cells were always intact and had a distinct appearance due to their yellowish granular composition. They had normal morphology and thickness and perform routine functions. When the worm was exposed to sublethal doses, there was a loss of cellular integrity and cell disintegration. Significant vacuolization was seen and villi were eroded and disintegrated along with the internal muscle layer. Cellular debris can be seen at 60% LC50 (Group B). Since the disintegration of muscle fibre was severe at 80% LC50 (Group C), damage to chloragogen cells was more evident due to its attachment to fibres in case of a higher dose. Under the amendment the cells were condensed and fused at some points, affecting the gut wall partially and causing them to lose their regular structural organization. Both the amendment and the control showed comparable organization to control, indicating a reduction in oxidative stress induced by the fungicide.

## Conclusion

The use of pesticide mixtures in agricultural fields significantly alters the diversity and structure of ecosystems, influencing both direct interactions and secondary or additive effects on organisms and their ecosystem functions. These effects can propagate through interconnected food webs, affecting various trophic levels in both bottom-up and top-down directions. Copper oxychloride, a widely used fungicide, is applied as a foliar spray during the vegetation period to combat fungal infections. Its mechanism involves copper ions that damage fungal cells by disrupting cellular membranes, coagulating protoplasm, and inhibiting metabolic processes. However, this study has demonstrated that copper oxychloride also poses a toxicity risk to non-target organisms, such as earthworms, through metal accumulation and oxidative stress driven by excessive reactive oxygen species (ROS) production.

Earthworms, particularly *Eisenia fetida*, play a critical role in soil health, and their tolerance influences bioaccumulation and vermi-remediation processes. In this study, *Eisenia fetida*, a key species, showed no mortality when exposed to copper oxychloride coated with iron nanoparticles. This suggests that the nanoparticle coating effectively reduced the acute toxicity of copper oxychloride. Although copper accumulation was detected in earthworms, it was lower than expected, indicating their regulatory mechanisms help mitigate accumulation.

The transport, behavior, and ecotoxicity of pesticides in soil are influenced by complex interactions, and soil toxicity investigations are challenging due to the varying stability and sorptive behavior of pesticides. Despite limited information on the effects of nanoparticle-coated pesticides on ecosystem health, particularly regarding soil invertebrates and plant stress responses, this study provides critical insights. This study did not observe mortality in earthworms exposed to coated NPs. The finding indicates that coated NPs (at least up to 1: 0.75 LC50 and NPs) are not acutely toxic but can potentially induce physiological and biochemical consequences in earthworms. To comprehend these subcellular impacts, *Eisenia fetida*, a regularly used terrestrial toxicity test species, was subjected to several molecular and cellular repercussions studies. *Eisenia fetida* exposed to fungicide-coated nanoparticles were compared to *Eisenia fetida* subjected to fungicide; this comparison allowed for an assessment of the degree to which toxicity is generated by the fungicide-coated vs. free fungicide. Understanding the bioaccumulation process is crucial when assessing the dangers and risks associated with pesticides.

## Data Availability

The data is contained within the article.

## References

[1] Source http://ppqs.gov.in/statistical-database?page=2

[2] Source-FAOSTAT, 2023. https://www.fao.org/faostat/en/#compare

[3] Alves, P. R. L., Cardoso, E. J., Martines, A. M., Sousa, J. P. & Pasini, A. Earthworm ecotoxicological assessments of pesticides used to treat seeds under tropical conditions. *Chemosphere* **90**(11), 2674–2682 (2013). 23261124 10.1016/j.chemosphere.2012.11.046

[4] Omar, H. M., Ibraheim, Z. Z., El-Shimy, N. A. & Ali, R. S. Anti-inflammatory, antipyretic and antioxidant activities of the earthworms extract. *J. Biol. Earth Sci.* **2**(1), 10–17 (2012).

[5] Rajiv, P. & Vanathi, P. Effect of *Parthenium* based vermicompost and zinc oxide nanoparticles on growth and yield of *Arachis hypogaea* L. in zinc deficient soil. *Biocatal. Agric. Biotechnol.* **13**, 251–257. 10.1016/j.bcab.2018.01.006 (2018).

[6] Bhuyan, T., Mishra, K., Khanuja, M., Prasad, R. & Varma, A. Biosynthesis of zinc oxide nanoparticles from *Azadirachta indica* for antibacterial and photocatalytic applications. *Mater. Sci. Semiconduct. Process.* **32**, 55–61. 10.1016/j.mssp.2014.12.053 (2015).

[7] Badmus, K. O., Coetsee-Hugo, E., Swart, H. & Petrik, L. Synthesis and characterisation of stable and efficient nano zero valent iron. *Environ. Sci. Pollut. Res.* **25**(24), 23667–23684. 10.1007/s11356-018-2119-7 (2018). 10.1007/s11356-018-2119-7 29748806

[8] Nahak, G. & Sahu, R. K. Evaluation of antioxidant activity of flower and seed oil of *Azadirachta indica* A. juss. *J. Appl. Nat. Sci.* **3**(1), 78–81 (2011).

[9] Dhama, K. et al. A comprehensive review on chemical profile and pharmacological activities of *Ocimum basilicum*. *Food Reviews Int.* 1–29. 10.1080/87559129.2021.1900230 (2021).

[10] Pattanayak, M. & Nayak, P. L. Green synthesis and characterization of zero valent iron nanoparticles from the leaf extract of *Azadirachta indica* (neem). *World J. Nano Sci. Technol.* **2**(1), 06–09 (2013).

[11] Wang, T., Jin, X., Chen, Z., Megharaj, M. & Naidu, R. Green synthesis of Fe nanoparticles using eucalyptus leaf extracts for treatment of eutrophic wastewater. *Sci. Total Environ.* **466–467**, 210–213. 10.1016/j.scitotenv.2013.07.022 (2014). 10.1016/j.scitotenv.2013.07.022 23895784

[12] Barrios, A. C. et al. Effects of uncoated and citric acid coated cerium oxide nanoparticles, bulk cerium oxide, cerium acetate, and citric acid on tomato plants. *Sci. Total Environ.* **563**, 956–964. 10.1016/j.scitotenv.2015.11.143 (2016). 26672385 10.1016/j.scitotenv.2015.11.143

[13] Edwards, C. A. & Bohlen, P. J. *Biology and Ecology of Earthworms*, Vol. 3 (Springer Science & Business Media, 1996).

[14] Bergknut, M. et al. Comparison of techniques for estimating PAH bioavailability: Uptake in *Eisenia fetida*, passive samplers and leaching using various solvents and additives. *Environ. Pollut.* **145**(1), 154–160 (2007). 16713049 10.1016/j.envpol.2006.03.052

[15] OECD, T. N. 207: Earthworm, acute toxicity tests. *OECD Guidelines for the Testing of Chemicals, Section*, *2* (1984).

[16] ISO (International Organization for Standardization). Effects of pollutants on earthworms (*Eisenia fetida*). Part 1: Determination of acute toxicity using artificial soil substrate- No. 11268-1. Geneva (1993).

[17] ISO (International Organization for Standardization). Effects of pollutants on earthworms (*Eisenia fetida*). Part 2: Determination of acute toxicity using artificial soil substrate- No. 11268-2. Geneva (1998).

[18] Garcia, M. *Effects of Pesticides on soil Fauna: Development of Ecotoxicological test Methods for Tropical Regions*, Vol. 19 (Cuvillier, 2004).

[19] Wang, Q. Y., Sun, J. Y., Xu, X. J. & Yu, H. W. Integration of chemical and toxicological tools to assess the bioavailability of copper derived from different copper-based fungicides in soil. *Ecotoxicol. Environ. Saf.* **161**, 662–668. 10.1016/j.ecoenv.2018.06.041 (2018). 29935430 10.1016/j.ecoenv.2018.06.041

[20] Song, Y. et al. DNA damage and effects on antioxidative enzymes in earthworm (*Eisenia foetida*) induced by atrazine. *Soil Biol. Biochem.* **41**(5), 905–909 (2009).

[21] Wang, Y. et al. Toxicity assessment of 45 pesticides to the epigeic earthworm *Eisenia fetida*. *Chemosphere* **88**(4), 484–491 (2012). 22459421 10.1016/j.chemosphere.2012.02.086

[22] Bradford, M. M. A rapid and sensitive method for the quantitation of microgram quantities of protein utilizing the principle of protein-dye binding. *Anal. Biochem.* **72**(1–2), 248–254 (1976). 942051 10.1016/0003-2697(76)90527-3

[23] Kochba, J., Lavee, S. & Spiegel-Roy, P. Differences in peroxidase activity and isoenzymes in embryogenic ane non-embryogenic ‘Shamouti’orange ovular callus lines. *Plant Cell Physiol.* **18**(2), 463–467 (1977).

[24] Goven, A. J., Chen, S. C., Fitzpatrick, L. C. & Venables, B. J. Lysozyme activity in earthworm (*Lumbricus terrestris*) coelomic fluid and coelomocytes: Enzyme assay for immunotoxicity of xenobiotics. *Environ. Toxicol. Chem. Int. J.* **13**(4), 607–613 (1994).

[25] Fourie, F., Reinecke, S. A. & Reinecke, A. J. The determination of earthworm species sensitivity differences to cadmium genotoxicity using the comet assay. *Ecotoxicol. Environ. Saf.* **67**(3), 361–368 (2007). 17173970 10.1016/j.ecoenv.2006.10.005

[26] Zhang, Q., Saleem, M. & Wang, C. Effects of biochar on the earthworm (*Eisenia foetida*) in soil contaminated with and/or without pesticide mesotrione. *Sci. Total Environ.* **671**, 52–58. 10.1016/j.scitotenv.2019.03.364 (2019). 30927727 10.1016/j.scitotenv.2019.03.364

[27] Cardiff, R. D., Miller, C. H. & Munn, R. J. Manual hematoxylin and eosin staining of mouse tissue sections. *Cold Spring Harbor Protocols* **2014**(6), pdb-prot073411. (2014). 10.1101/pdb.prot073411 24890205

[28] GraphPad Prism version 8.0.0 for Windows, GraphPad Software, San Diego, California USA, www.graphpad.com.

[29] Nasiri, H., Dorranian, D. & Sari, A. H. Green laser assisted gold-iron oxide nanocomposite production. *Radiat. Eff. Defects Solids* **177**(3–4), 277–293 (2022).

[30] Ebrahiminezhad, A., Taghizadeh, S., Ghasemi, Y. & Berenjian, A. Green synthesized nanoclusters of ultra-small zero valent iron nanoparticles as a novel dye removing material. *Sci. Total Environ.* **621**, 1527–1532. 10.1016/j.scitotenv.2017.10.076 (2018). 29054616 10.1016/j.scitotenv.2017.10.076

[31] Das, J. & Dhar, S. S. *Camellia sinensis* mediated synthesis of zero valent iron nanoparticles and study of their efficacy in dye degradation and antibacterial activity. *Int. J. Environ. Anal. Chem.* **102**(18), 7241–7254 (2022).

[32] Thiruppathi, M., Leeladevi, K., Ramalingan, C., Chen, K. C. & Nagarajan, E. R. Construction of novel biochar supported copper tungstate nanocomposites: A fruitful divergent catalyst for photocatalysis and electrocatalysis. *Mater. Sci. Semiconduct. Process.* **106**, 104766. 10.1016/j.mssp.2019.104766 (2020).

[33] Venter, J. M. & Reinecke, A. J. The life-cycle of the compost worm. *Afr. Zool.* **23**(3), 161–165 (1988).

[34] Kumar, S. & Singh, S. M. Artificial soil test of phorate* Eisenia fetida*. *J. Environ. Anal. Toxicol.* **6**(391), 2161–0525 (2016).

[35] Hu, M. et al. Behavior of imidazolinone herbicide enantiomers in earthworm-soil microcosms: Degradation and bioaccumulation. *Sci. Total Environ.* **707**, 135476. 10.1016/j.scitotenv.2019.135476 (2020). 31771851 10.1016/j.scitotenv.2019.135476

[36] Zhang, M. et al. Variations of earthworm gut bacterial community composition and metabolic functions in coastal upland soil along a 700-year reclamation chronosequence. *Sci. Total Environ.* **804**, 149994. 10.1016/j.scitotenv.2021.149994 (2022). 34798714 10.1016/j.scitotenv.2021.149994

[37] Morgan, J. E. & Morgan, A. J. The effect of lead incorporation on the elemental composition of earthworm (Annelida, Oligochaeta) chloragosome granules. *Histochemistry* **92**(3), 237–241 (1989). 2777641 10.1007/BF00500924

[38] Spurgeon, D. J. & Hopkin, S. P. Effects of metal-contaminated soils on the growth, sexual development, and early cocoon production of the earthworm *Eisenia fetida*, with particular reference to zinc. *Ecotoxicol. Environ. Saf.* **35**(1), 86–95 (1996). 8930509 10.1006/eesa.1996.0085

[39] Zhang, Q. et al. Oxidative stress and lipid peroxidation in the earthworm *Eisenia fetida* induced by low doses of fomesafen. *Environ. Sci. Pollut. Res.* **20**(1), 201–208. 10.1007/s11356-012-0962-5 (2013). 10.1007/s11356-012-0962-5 22585392

[40] Li, M. et al. Influences and mechanisms of nanoparticles on pentachloronitrobenzene accumulation by earthworms. *Environ. Sci. Pollut. Res.* **28**(37), 51471–51479 (2021). 10.1007/s11356-021-14368-6 33983610

[41] Hinton, D. E. et al. Histopathologic biomarkers. In *Biomarkers* 155–210 (CRC, 2018).

